# Genetic Diversity of the *Cryptococcus* Species Complex Suggests that *Cryptococcus gattii* Deserves to Have Varieties

**DOI:** 10.1371/journal.pone.0005862

**Published:** 2009-06-10

**Authors:** Popchai Ngamskulrungroj, Felix Gilgado, Josiane Faganello, Anastasia P. Litvintseva, Ana Lusia Leal, Kin Ming Tsui, Thomas G. Mitchell, Marilene Henning Vainstein, Wieland Meyer

**Affiliations:** 1 Molecular Mycology Research Laboratory, Centre for Infectious Diseases and Microbiology, Westmead Millennium Institute, Westmead Hospital, Westmead, New South Wales, Australia; 2 The University of Sydney Western Clinical School, Faculty of Medicine, University of Sydney, Sydney, New South Wales, Australia; 3 Faculty of Medicine, Siriraj Hospital, Mahidol University, Bangkok, Thailand; 4 Centro de Biotecnologia, Universidade Federal do Rio Grande do Sul, Porto Alegre, Rio Grande do Sul, Brazil; 5 Department of Molecular Genetics and Microbiology, Duke University Medical Center, Durham, North Carolina, United States of America; Duke University, United States of America

## Abstract

The *Cryptococcus* species complex contains two sibling taxa, *Cryptococcus neoformans* and *Cryptococcus gattii*. Both species are basidiomycetous yeasts and major pathogens of humans and other mammals. Genotyping methods have identified major haploid molecular types of *C. neoformans* (VNI, VNII, VNB and VNIV) and of *C. gattii* (VGI, VGII, VGIII and VGIV). To investigate the phylogenetic relationships among these haploid genotypes, we selected 73 strains from 2000 globally collected isolates investigated in our previous typing studies, representing each of these genotypes and carried out multigene sequence analyses using four genetically unlinked nuclear loci, *ACT1, IDE, PLB1* and *URA5*. The separate or combined sequence analyses of all four loci revealed seven clades with significant support for each molecular type. However, three strains of each species revealed some incongruence between the original molecular type and the sequence-based type obtained here. The topology of the individual gene trees was identical for each clade of *C. neoformans* but incongruent for the clades of *C. gattii* indicating recent recombination events within *C. gattii*. There was strong evidence of recombination in the global VGII population. Both parsimony and likelihood analyses supported three major clades of *C. neoformans* (VNI/VNB, VNII and VNIV) and four major clades of *C. gattii* (VGI, VGII, VGIII and VGIV). The sequence variation between VGI, VGIII and VGIV was similar to that between VNI/VNB and VNII. *MATa* was for the first time identified for VGIV. The VNIV and VGII clades are basal to the *C. neoformans* or the *C. gattii* clade, respectively. Divergence times among the seven haploid monophyletic lineages in the *Cryptococcus* species complex were estimated by applying the hypothesis of the molecular clock. The genetic variation found among all of these haploid monophyletic lineages indicates that they warrant varietal status.

## Introduction

The *Cryptococcus* species complex includes two basidiomycetous encapsulated yeast species, *Cryptococcus neoformans*, an opportunistic pathogen, and *Cryptococcus gattii,* a primary pathogen. Both species are the most common fungal agents of infection of the central nervous system [1]. Two varieties of *C. neoformans* are recognized, *C. neoformans* var. *grubii* (serotype A), which is found worldwide, and *C. neoformans* var. *neoformans* (serotype D), which occurs mainly in Europe and South America [1], [2], [3]. *C. gattii* [4], was previously known as *C. neoformans* var. *gattii* (serotype B and C), and thought to be restricted to tropical and subtropical zones [2], [5] until a recent outbreak of cryptococcosis occurred on Vancouver Island, Canada, which has expanded the range of this yeast to temperate regions [6].

In recent years, an array of molecular studies, including PCR-fingerprinting [7], Amplified Fragment Length Polymorphisms (AFLP) analysis [8], and analysis of the orotidine monophosphate pyrophosphorylase (*URA5*) [9] and phospholipase (*PLB1*) [10] genes by Restriction Fragment Length Polymorphism (RFLP), have grouped all globally obtained strains into eight distinct molecular types: VNI ( = AFLP1) and VNII ( = AFLP1A and AFLP1B) (*C. neoformans* var. *grubii,* serotype A); VNIV ( = AFLP2) (*C. neoformans* var. *neoformans*, serotype D); VNIII ( = AFLP3) (hybrid, serotype AD); and VGI ( = AFLP4), VGII ( = AFLP6), VGIII ( = AFLP5) and VGIV ( = AFLP7), all corresponding to serotypes B and C (*C. gattii*), indicating that they have evolved independently in parallel. Similar sub-groups, representing the same level of genetic heterogeneity have been found in subsequent sequencing studies [11], [12], [13], [14], [15]. The degree of variation among these molecular types indicated that the varieties and species are genetically distinct and undergoing evolutionary divergence [11]. AFLP and MLST analyses recently identified strains closely related to the VNI group as being unique to Botswana, and suggested for those a new molecular type, VNB [15]. In addition, hybrid strains of *C. gattii* have been reported but no specific molecular type(s) were designated [8, Trilles *et al.*, unpublished data]. More recently, naturally occurring hybrids between *C. neoformans* and *C. gattii* were reported as DaBα AFLP8 [16].

Here, we investigated the phylogenetic relationships of the species and varieties of the *Cryptococcus* species complex to test the hypothesis that each haploid molecular type is monophyletic. We excluded the molecular type VNIII, which contains hybrid individuals of serotype AD. The phylogenetic analysis is based on comparing patterns of sequence variation across four unlinked genes involved in housekeeping, production of secreted enzymes and virulence: actin (*ACT1*) [17], orotate-phosphoribosyl transferase (*URA5*) [3], phospholipase B (*PLB1*) [18] and the gene encoding a 110-kDa neutral metalloendopeptidase (*IDE*) involved in degradation of insulin in humans and mammals [19]. The first three genes were chosen because they are polymorphic among various groups of the *Cryptococcus* species complex [3], [9], [10], [17]. IDE is novel for this investigation. This gene was chosen because it is polymorphic between both *Cryptococcus* species [19]. In addition, this gene is conserved across a wide range of organisms [20], [21], [22], which makes it a candidate locus to be used for studying inter-species phylogenetic relationships [23]. The herein presented multigene sequencing data revealed seven major haploid lineages within the *Cryptococcus* species complex and provide further evidence to consider these major molecular types as individual varieties, if not species.

## Results

### Strains

From a collection of 2000 cryptococcal strains previously analyzed, we selected ten strains each of the haploid molecular types VNI, VNII, VNIV, VGI, VGIII and VGIV, and 13 strains of VGII, representing six continents [9], [15], [24], [25], [26, Meyer *et al.* unpublished data] (Table 1 and 2). Only South America contained strains of every molecular type. In addition, four VNB strains, which until now had only been reported from Africa [15], were used to represent this new molecular type.

**Table 1:** List of strains used in this study, including general strain information, serotype (ST), mating type (MAT), molecular type (MT) and the allele assignment for the four genes used in the multigene analysis.

Isolates	WM No.	Country	Source	ST	MAT	MT	References	Allele Assignment			
								*ACT1*	*URA5*	*PLB1*	*IDE*
***Cryptococcus neoformans*** ** var. ** ***grubii***											
ATCC 90112	WM 419	USA	CLIN	A	alpha	VNI	[74]	1	1	1	1
M27049	WM 2573	South Africa	CLIN	-	alpha	VNI	This study	2	1	2	1
WM 721	WM 721	India	ENV	A	alpha	VNI	This study	3	1	2	1
WM 148^R^	WM 148	Australia	CLIN	A	alpha	VNI	[9]	3	2	16	1
RV 59369	WM 1416	Belgium	ENV	A	alpha	VNI	This study	1	1	1	2
NIH 193	WM 1421	USA	ENV	A	alpha	VNI	This study	2	1	1	1
LA 26	WM 1641	Mexico	ENV	A	alpha	VNI	[9]	2	3	15	1
LA 182	WM 1897	Spain	CLIN	A	alpha	VNI	[9]	2	1	15	1
LA 264	WM 1742	Chile	CLIN	A	alpha	VNI	[9]	2	1	1	1
LA 473	WM 1948	Colombia	CLIN	A	alpha	VNI	[9]	1	1	1	1
H99	WM 846	USA	CLIN	A	alpha	VNI	[75]	-	-	-	-
JG-02	WM 2529	USA	CLIN	-	alpha	VNII	This study	5	5	5	5
M27053	WM 2577	South Africa	CLIN	A	alpha	VNII	This study	4	4	3	18
PR-101	WM 1352	India	CLIN	-	alpha	VNII	This study	6	5	5	19
UON 11536	WM 1462	South Africa	CLIN	-	alpha	VNII	This study	6	5	5	5
WM626^R^	WM 626	Australia	CLIN	A	alpha	VNII	[9]	6	7	5	6
Hamden C3-1	WM 1408	Brazil	ENV	A	alpha	VNII	[8]	7	21	4	3
RV 58146	WM 1412	Zaire	ENV	A	alpha	VNII	[12]	5	6	5	4
LA 146	WM 553	Brazil	ENV	A	alpha	VNII	[9]	6	5	5	5
LA 404	WM 1816	Mexico	CLIN	A	alpha	VNII	[9]	6	5	5	5
LA 511	WM 1986	Colombia	CLIN	A	alpha	VNII	[9]	7	21	4	3
bt1		Botswana	CLIN	-	-	VNB	[15]	-	-	-	-
bt22		Botswana	CLIN	-	-	VNB	[15]	-	-	-	-
bt31		Botswana	CLIN	-	-	VNB	[15]	-	-	-	-
bt131		Botswana	CLIN	-	-	VNB	[15]	-	-	-	-
***Cryptococcus neoformans*** **var.** ***Neoformans***											
WM 629^R^	WM 629	Australia	CLIN	D	alpha	VNIV	[9]	13	10	6	8
RKI-M186/99	WM 04.174	Germany	CLIN	D	alpha	VNIV	This study	9	11	17	7
RKI-M318/90	WM 04.172	Germany	CLIN	D	alpha	VNIV	This study	9	11	17	7
B-3501	WM 2242	USA	CLIN	D	alpha	VNIV	[76]	12	23	8	20
CBS 7816		Thailand	ENV	D	a	VNIV	[12]	12	22	8	7
LA268	WM 04.168	Chile	CLIN	D	alpha	VNIV	[9]	12	9	7	9
JEC 20	WM 01.126	USA	NA	D	a	VNIV	[77]	12	22	8	7
JEC 21	WM 01.127	USA	NA	D	alpha	VNIV	[77]	11	22	8	7
KRIMM 2	WM 02.142	Russia	CLIN	-	alpha	VNIV	This study	10	8	6	21
LA262	WM 1740	Chile	CLIN	D	alpha	VNIV	[9]	8	10	6	8
***Cryptococcus gattii***											
LA1	WM 1616	Mexico	CLIN	B	alpha	VGI	[9]	15	16	9	15
503 2738	WM 1251	Papua New Guinea	CLIN	B	alpha	VGI	[78]	16	16	11	14
WM 179^R^	WM 179	Australia	CLIN	B	alpha	VGI	[9]	15	17	9	14
Joe	WM 1243	Papua New Guinea	CLIN	B	alpha	VGI	[78]	15	16	11	14
MC-S-022	WM 2634	Thailand	CLIN	B	alpha	VGI	[79]	17	31	23	16
TP 0688	WM 727	USA	ENV	B	alpha	VGI	This study	15	16	11	14
TP 1414	WM 2540	New Zealand	VET	B	alpha	VGI	This study	15	16	9	14
LA175	WM 1899	Spain	CLIN	B	alpha	VGI	[9]	14	19	24	15
LA 564	WM 2039	Colombia	CLIN	B	alpha	VGI	[9]	15	16	18	14
F 2863	WM 02.204	Canada	VET	B	alpha	VGI	[6]	14	18	25	24
WM 1008	WM 1008	Australia	ENV	-	alpha	VGII	[78]	20	12	13	12
WM 178^R^	WM 178	Australia	CLIN	B	alpha	VGII	[9]	21	25	33	13
MC-S-239	WM 06.7	Thailand	CLIN	B	alpha	VGII	[79]	18	13	31	12
RAM 002	WM 03.27	Australia	ENV	-	alpha	VGII	[78]	19	12	13	12
CBS 7750	WM 06.13	USA	ENV	B	alpha	VGII	[6]	19	13	31	12
LA 43	WM 04.191	Uruguay	ENV	B	alpha	VGII	[9]	21	12	30	12
LA 84	WM 477	Brazil	CLIN	-	alpha	VGII	[9]	18	24	28	12
CDC R369	WM 02.46	Canada	CLIN	B	alpha	VGII	[6]	18	13	31	12
NIH 444	WM 02.81	USA	CLIN	B	alpha	VGII	[80]	18	13	31	12
RB52	WM 02.317	Canada	ENV	B	alpha	VGII	[6]	18	12	13	12
AV 55	WM 05.77	Greece	CLIN	B	a	VGII	[81]	21	14	14	12
AV 54W	WM 05.75	Greece	CLIN	B	alpha	VGII	[81]	21	15	13	12
AV 54S	WM 05.76	Greece	CLIN	B	alpha	VGII	[81]	21	15	13	12
WM 175^R^	WM 175	USA	ENV	B	alpha	VGIII	[9]	22	26	26	11
CN043	WM 2423	New Zealand	CLIN	-	a	VGIII	[78]	23	29	19	22
TP 0686	WM 728	USA	ENV	B	alpha	VGIII	This study	22	26	26	11
TP 0689	WM 161	USA	ENV	B	alpha	VGIII	This study	22	28	26	11
TP 0696	WM 726	USA	ENV	B	alpha	VGIII	This study	22	26	26	11
UCLA 380C	WM 1665	USA	N	C	alpha	VGIII	[76]	23	30	22	11
LA 290	WM 1699	Paraguay	CLIN	-	alpha	VGIII	[9]	23	30	10	11
LA 382	WM 1846	Venezuela	CLIN	C	alpha	VGIII	[9]	23	30	19	11
LA 622	WM 2176	Colombia	CLIN	B	a	VGIII	[9]	22	26	21	11
LA 644	WM 2158	Colombia	ENV	C	alpha	VGIII	[9]	23	30	19	11
WM 779^R^	WM 779	South Africa	VET	C	alpha	VGIV	[9]	24	32	27	23
B-5748	WM 2364	India	CLIN	B	alpha	VGIV	This study	24	32	27	23
B-5742	WM 2363	India	CLIN	B	alpha	VGIV	This study	24	32	27	23
M27055	WM 04.20	South Africa	CLIN	C	alpha	VGIV	This study	24	32	12	23
M27056	WM 2579	South Africa	CLIN	-	alpha	VGIV	This study	24	32	12	23
V00709	WM 780	South Africa	CLIN	C	alpha	VGIV	This study	24	32	12	23
V00869	WM 2876	South Africa	CLIN	C	alpha	VGIV	This study	24	32	29	23
LA 390	WM 1802	Mexico	CLIN	-	a	VGIV	[9]	25	20	32	17
LA 392	WM 1804	Mexico	CLIN	-	a	VGIV	[9]	25	20	32	17
LA 568	WM 2041	Colombia	CLIN	B	alpha	VGIV	[9]	26	27	20	10
**Outgroups**											
***Filobasidiella depauperata***											
CBS7841		Canada	ENV	NA	NA	NA	[82]	-	-	-	-
***Cryptococcus albidus***											
CBS142	WM 773	Japan	ENV	NA	NA	NA	[83]	-	-	-	-

### Mating type analysis

**Figure 1:**
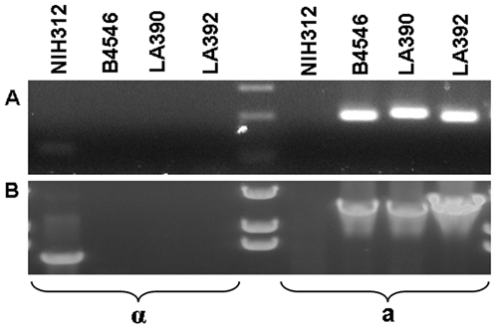
Mating type specific PCR amplification of the two mating type a VGIV strains LA390 and LA392. A) primers MFαU and MFαL: MFa2U and MFa2L; B) primers SXI1αF and SXI1αR: SXI2aF and SXI2aR.

Primers specific for the MFα and MF**a** pheromone confirmed that *MATα* was predominant (66 out of 73 strains). Seven strains possessed the *MAT* ***a*** allele: 2 VNIV, 1 VGI, 2 VGIII and 2 VGIV strains (Table 1). Non-specific amplicons were produced by *MAT* ***a*** strains of VGIII and VGIV (data not shown). Therefore, bands of a length corresponding to the *MAT* ***a*** amplicon were sequenced, and BlastN searches revealed 98% sequence similarity with the mating pheromone **a** 2 (MFa2) gene of the *C. gattii* strain E566 (GenBank accession No. AY710429). The two VGIII strains CN043 and LA 622 as well as the two VGIV strains LA 390 and LA 392 were accordingly designated as mating type **a** (GenBank Accession No. EU408654 – EU408657) (Table 1 and 2). To our knowledge, this is the first report of the *MAT* **a** mating type in strains of the molecular type VGIV. To confirm this finding we used primers specific for the *SXI1α* and *SXI2* ***a*** genes and obtained the same result as above (Figure 1).

**Table 2:** Geographic distribution and mating type data of all studied isolates.

Molecular type	Continent						Total^*^
	Africa	Asia	Australia	Europe	North America	South America	
VNI	1	1	1	2	2	3	10 (0)
VNII	3	1	1	0	1	4	10 (0)
VNIV	0	1 (1)		3	3 (1)	2	10 (2)
VGI	0	1	4	1	2	2	10 (0)
VGII	0	1	3	3 (1)	4	2	13 (1)
VGIII	0	0	1 (1)	0	5	4 (1)	10 (2)
VGIV	5	2	0	0	0	3 (2)	10 (2)
**Total**	**8**	**9**	**14**	**15**	**16**	**21**	**83 (7)**

### Mating

**Figure 2:**
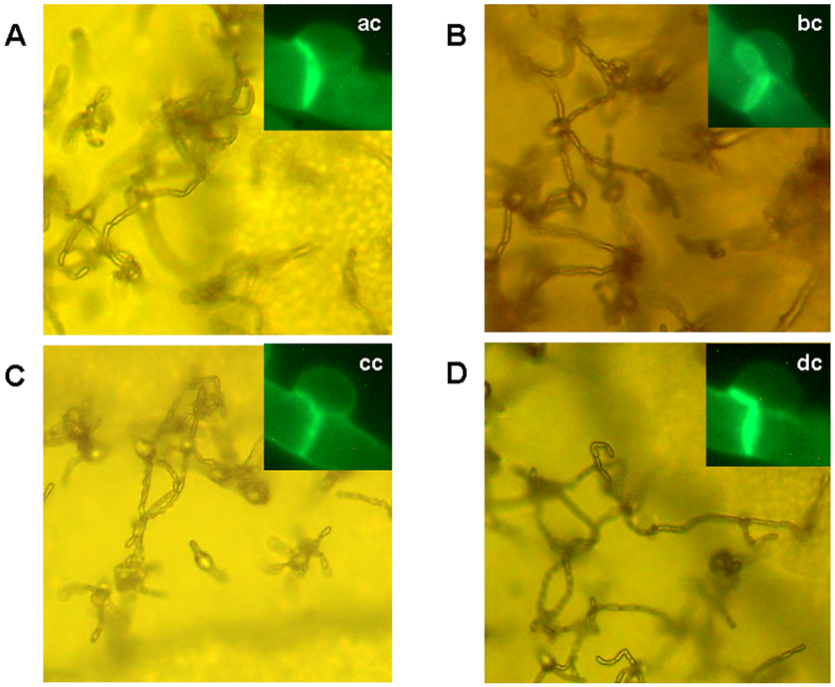
Mating reaction on V8 media of the two mating type a VGIV strains. A) LA390×NIH312; B) LA390×JF101; C) LA392×NIH312; D) LA392×JF101. All strains revealed typical bacilli-shape basidiospores and clamp connections (ac, bc, cc and dc).

Mating of the two VGIV strains, LA 390 and LA 392, with the *MATα* reference strain of *C. gattii*, serotype C, NIH312 [27] and the *crg1*α mutant derivatives *MATα* supermater tester strain, JF101 [28] produced dikaryotic hyphae with fused clamp connections, basidia and bacilli-shaped basidiospores (Figure 2). However, the strains LA 390 and LA 392 failed to mate with the *MAT* ***a*** reference tester strains B4546 [29] and JF109 [28] (data not shown). Therefore, both strains, LA 390 and LA 392, were confirmed to possess the *MAT* ***a*** mating type allele. No haploid fruiting was observed when the samples were incubated alone on V8 juice agar.

### Sequencing data

Four independent genetic loci, *ACT1, URA5, PLB1* and *IDE*, were sequenced and all sequences were deposited in GenBank under the following accession numbers: *ACT1* (EU408478–408550); *URA5* (EU399554–EU399626); *PLB1* (EU408624–EU408653, EU408658–408700) and *IDE* (EU408551–EU408623). The outgroup sequences have the following GenBank accession numbers: *F. depauperata ACT1* (EU399627) and *URA5* (EU399628); *C. albidus ACT1* (EU399629).

### Phylogenetic analyses of individual and combined loci

Maximum parsimony and Bayesian methods were used to analyze phylogenetic relationships among the 73 cryptococcal strains selected, using four independent genetic loci, *ACT1, URA5, PLB1* and *IDE*.

The four loci of the intron-excluded and intron-included datasets contained a total of 564 and 834 parsimoniously informative characters, respectively. A hypervariable region containing poly-T was found in the intron of the *PLB1* gene of some VGII strains. This region was excluded from the analysis due to sequencing problems. A heuristic search of the *ACT1* gene sequences resulted in two maximum parsimony trees (Length 351, CI 0.855, RI 0.957); the *URA5* gene produced 1152 maximum parsimony trees (Length 208, CI 0.897, RI 0.973); the *PLB1* gene produced six maximum parsimony trees (Length 395, CI 0.922, RI 0.994); and the *IDE* gene produced 24 maximum parsimony trees (Length 113, CI 0.912, RI 0.993). Character information and substitution models of each locus are presented in Table 3. Bayesian analyses revealed topologies very similar or identical to those obtained using maximum parsimony. The topologies of each gene in both intron-excluded and intron-included datasets were identical or very similar (data not shown).

**Table 3:** Phylogenetic characters of the intron-excluded and intron-included data sets.

Locus	Character (intron-excluded)			Character (intron-included)			substitution model
	total	constant	parsimony informative	total	constant	parsimony informative	
***ACT1***	1124	848	177	1321	808	187	GTR+G
***URA5***	621	457	71	724	509	102	SYM+G
***PLB1***	1877	1549	309	2265	1819	423	HKY+G
***IDE***	581	483	85	684	549	122	K80+G*
**Combined**	4203	3337	564	4994	3685	834	N/A

Note: N/A = not applicable (partition of the dataset was used).

* The second best model was chosen by the ModelTest program since the best (TrN+I and TrN+G) could not be operated in the MrBayes program due to the limitation of this software.

To focus on the relationships among species and subgroups, and to avoid detecting incongruence among recombining strains within the lineages, a subset of strains was used for the analysis of combined data from four loci. This subset included a single representative strain from each of the major lineages identified by the single locus analyses (see Material and Methods for the strain numbers). Prior to the analysis, the genes were tested using incongruence length difference/partition homogeneity test (ILD/PHT), which revealed no significant incongruence among the loci, when a conservative threshold of P<0.0001 was used. Because ILD test is prone to type I errors of incorrectly rejecting the null hypothesis of congruence among the datasets, a conservative threshold of P<0.0001 is recommended for interpreting the results of this test [30]. In our case, the P value of the ILD test of the combined dataset was 0.002, indicating that the null hypothesis of congruence cannot be rejected [30].

**Figure 3:**
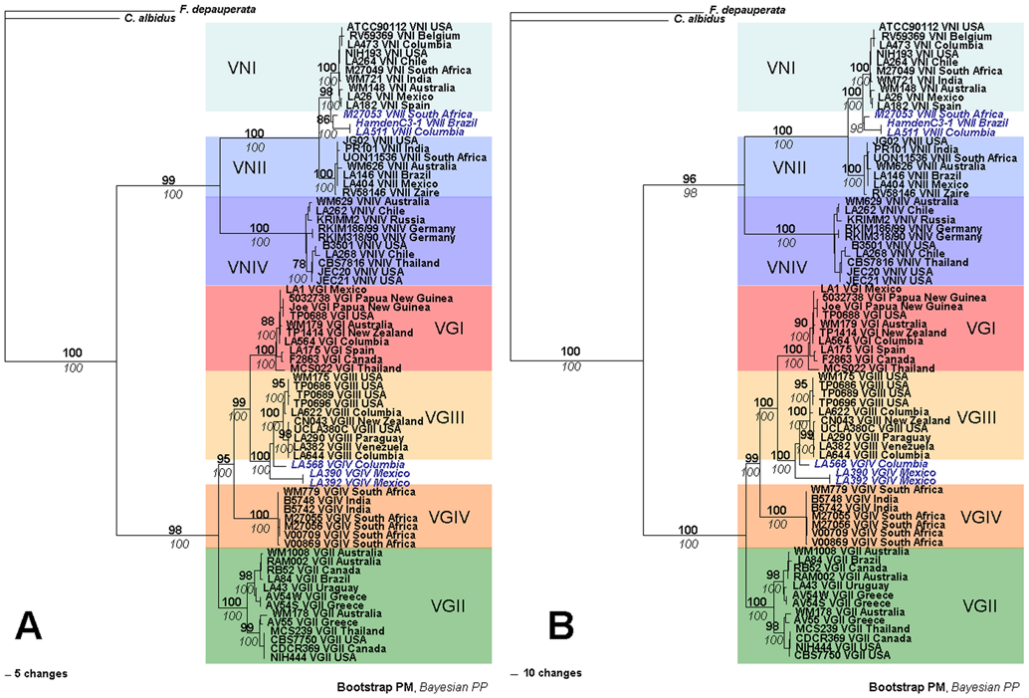
Combined genealogies of (A) the intron-excluded and (B) the intron-included datasets with separate substitution models for each partition. Parsimony bootstrap support above 75 is indicated in bold. Bayesian posterior probability above 95 is indicated italicized. The phylogenetic tree is rooted using *Filobasidiella depauperata* and *Cryptococcus albidus* as outgroups. The blue bold italic letters represent VNII-1 and VGIV-1 clades of *C. neoformans* and *C. gattii*, respectively.

A heuristic search of the combined loci found 110 maximum parsimony trees (Length 1089, CI 0.864, RI 0.984) without introns and 1023 maximum parsimony trees (Length 1706, CI 0.866, RI 0.984) with introns. Bayesian analyses of the combined dataset revealed that topologies were very similar or identical to those obtained using maximum parsimony. Bayesian analyses of the data, including both exons and introns as well as excluding the introns generated phylograms with identical topologies and comparable statistical support. Both analyses strongly support the monophyly of *C. neoformans* and *C. gattii* (Figure 3). As expected, the VNI and VNII molecular types, which represent *C. neoformans* var. *grubii* have a sibling relationship with the VNIV molecular type, representing *C. neoformans* var. *neoformans*. In addition VNIV is basal to VNI and VNII (Figure 3). Clades representing molecular types VGI, VGIII and VGIV are more closely related to one another than to the sibling VGII clade, which is positioned basal to them (Figure 3).

Unexpectedly, three *MATα* strains with the *URA5-*RFLP pattern of molecular type VNII, one each from South Africa (M27053), Brazil (HamdenC3-1) and Colombia (LA 511), clustered with the VNI clade and not with the expected VNII clade. Similarly, two *MAT* ***a*** strains from Mexico (LA 390 and LA 392) and one *MATα* strain form Colombia (LA 568) with the *URA5*-RFLP pattern of the molecular type VGIV, clustered with the VGIII clade. For convenience, these groups will be referred to as the “VNII-1 group” and the “VGIV-1 group”, respectively (Figure 3).

**Figure 4:**
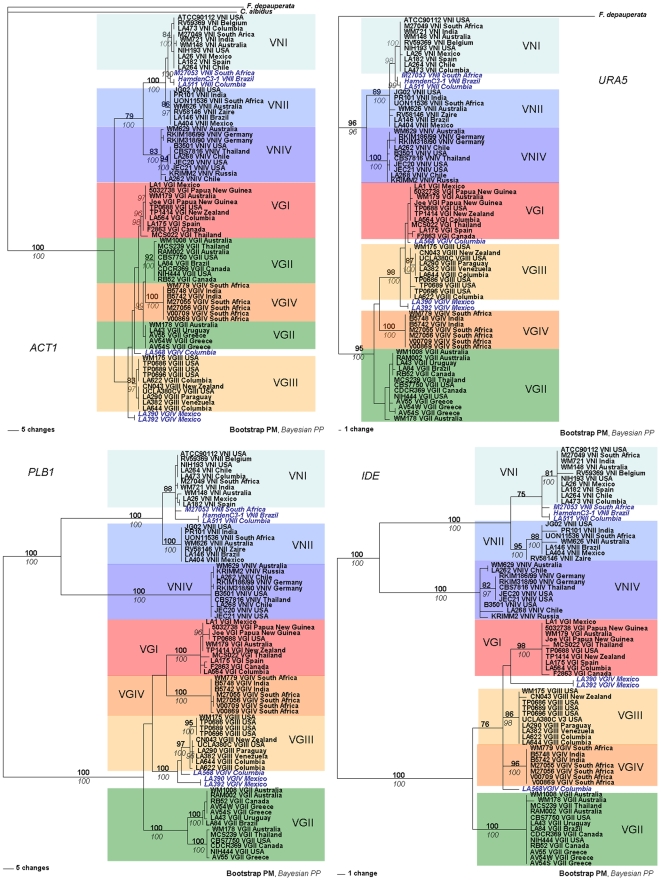
Gene genealogies of the four individual loci generated by Maximum Parsimony analysis. Parsimony bootstrap support above 75 is indicated in bold. Bayesian posterior probability above 95 is indicated italicized. Phylogenetic trees are unrooted. The blue bold italic letters represent VNII-1 and VGIV-1 clades of *C. neoformans* and *C. gattii*, respectively.

For the *C. neoformans* taxa, the topology of the individual loci never conflicted with the phylogram of the combined loci. The analysis of the two *C. neoformans* var. *grubii* clades (VNI and VNII) confirmed their sibling relationship with the *C. neoformans* var. *neoformans* clade (VNIV) (Figure 4). Conversely, the topologies of the individual loci of *C. gattii* conflicted with the phylogram of the combined loci. On the *PLB1, URA5* and *IDE* phylogenies, the VGI, VGIII and VGIV clades are clustered together and formed a sibling group with VGII. However, the phylogram generated from *ACT1* has a different topology in which, VGII strains are subdivided into two subclades. The deep branches of the *ACT1* phylogram did not achieve strong statistical support in the *C. gattii* clade, unlike the combined data set. However, both analyses generated high statistical support for the major clades, with the exception of the VGI and VGII clades of the *URA5* phylogram (Figure 4).

**Figure 5:**
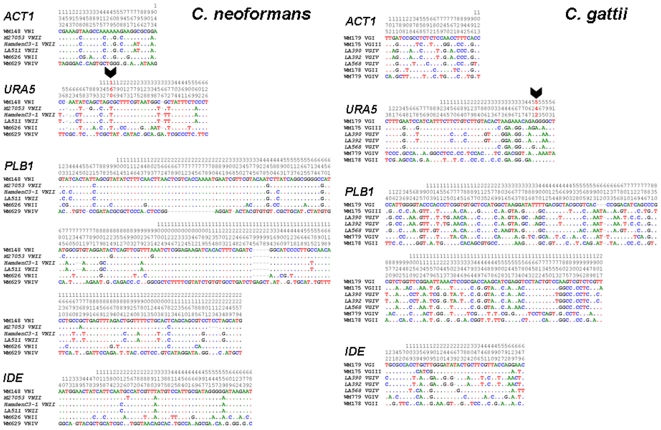
Variable sequence positions of all alignments of the VNII-1 and VGIV-1 strains revealed sequence similarity to VNI (ATCC90112 and WM148) and VGIII (WM175 and CN043) respectively. However, some parts of the sequence were similar to that of VNII (WM626 and RV58146) for the VNII-1 strains and VGIV (WM779 and M27056) for the VGIV-1 strains. Black thick arrows reveal one of the cutting sites for *URA5* RFLP giving VNII and VGIV patterns for the VNII-1 and VGIV-1 strains respectively.

The VNII-1 strains formed a monophyletic group, which was positioned between the VNI and VNII clades. Although Figure 3 and 4 suggest that the VNI-1 clade is more closely related to VNI, Figure 5 indicates that it has partial VNII sequence characteristics. The *URA5* sequence analysis of the VNII-1 isolates revealed that these isolates are missing the recognition site for *Sau*96I at position 160, which is a specific, identifying marker of VNI isolates (Figure 5), and the overall sequence similarity of the combined four loci showed that those isolates were more similar to VNI (99.41%) than VNII (98.91%) (Table 4).

**Table 4:** Sequence similarity matrix among the haploid molecular type clades of the *Cryptococcus* species complex.

	VNI	VNII	VNII-1	*VNII*	VNIV	VGI	VGII	VGIII	VGIV	VGIV-1	*VGIV*
VNI		*0.16*	*0.11*	*0.13*	*0.35*	*0.49*	*0.45*	*0.43*	*0.44*	*0.43*	*0.43*
VNII	98.98		*0.15*	*0.05*	*0.36*	*0.51*	*0.45*	*0.46*	*0.46*	*0.46*	*0.45*
VNII-1	99.41	98.91		*0.11*	*0.34*	*0.49*	*0.45*	*0.44*	*0.45*	*0.44*	*0.43*
***VNII***	99.11	99.63	99.16		*0.35*	*0.5*	*0.45*	*0.45*	*0.45*	*0.45*	*0.44*
VNIV	94.74	94.71	94.68	94.7		*0.45*	*0.42*	*0.44*	*0.43*	*0.45*	*0.42*
VGI	90.92	90.81	90.72	90.78	91.16		*0.24*	*0.19*	*0.23*	*0.19*	*0.19*
VGII	91.37	91.22	91.23	91.23	91.68	97.28		*0.24*	*0.19*	*0.24*	*0.18*
VGIII	91.18	91.15	91.00	91.11	91.40	98.07	97.40		*0.24*	*0.12*	*0.18*
VGIV	91.02	90.82	90.82	90.82	91.22	97.21	97.20	97.32		*0.22*	*0.07*
VGIV-1	90.91	90.84	90.73	90.81	91.13	97.75	97.16	98.87	97.06		*0.17*
***VGIV***	90.99	90.82	90.80	90.82	91.2	97.37	97.19	97.79	99.11	97.76	

* ***VNII*** = VNII+VNII-1; ***VGIV*** = VGIV+VGIV-1; numbers in italics designated values of standard errors.

Similarly, strains from the VGIV-1 group did not consistently form a monophyletic relationship with any of the *C. gattii* molecular type clades and were not supported by either analysis, except for the *PLB1* gene, whose topology was similar to that of the combined loci (Figure 3 and 4). However, the sequence analysis of the four genes revealed VGIII strains that contained integrated parts of VGIV sequences, which resemble the intermediate position of the VNII-1 strains of *C. neoformans* (Figure 5). The *URA5* sequence analysis of the VGIV-1 isolates revealed that those isolates share the recognition site for *Sau*96I with VGIV at position 542, which lead to their identification as VGIV (Figure 5), but the sequence similarity varied specifically for each of the four loci we investigated. The overall similarity to VGIII was 98.87% and to VGIV, 97.06%, resulting in an exchange of the position of those isolates in the individual gene trees (Table 4).

### Recombination and clonality

To determine the extent of clonality and recombination in populations of different molecular types, we used three different tests of linkage disequilibrium, (i) the incongruence length difference/partition homogeneity test (ILD/PHT), (ii) two measures of index of association (I_A_ and rBarD), and (iii) the phylogenetic incompatibility test. Since clonal reproduction can mask the effect of recombination, we prepared two different databases: one included all strains of each molecular type and the other included the clone corrected data of each molecular type from which identical genotypes were removed.

The ILD/PHT test showed similar results for each dataset, including and excluding individual genotypes (clone corrected). With the exception of VGII, the null hypothesis of clonality was not rejected. VGII showed incongruence in the phylogenies that allowed the rejection of clonality (P<0.0001).

In the second test we used two indexes of association measures, I_A_ and rBarD. These tests are expected to be zero if populations are freely recombining and greater than zero if there is association between alleles (clonality). In the I_A_ and rBarD tests including all isolates, the null hypothesis of recombination was rejected in all molecular types with the exception of VNI, which had the lowest value (Table 5). However, when the clonally corrected dataset was used, the I_A_ and rBarD showed clear evidence of recombination in the molecular types VNI, VGII and VGIII (Table 5) and confirmed the predominance of clonal reproduction among the other molecular types (VNII, VNIV, VGI and VGIV).

**Table 5:** Multilocus linkage disequilibrium analyses in each of the haploid molecular types of the *Cryptococcus* species complex.

Population	No. of isolates	No. of haplotypes	All isolates			Haplotypes only		
			I_A_	rBarD	PhI	I_A_	rBarD	PhI
VNI	10	8	0.1595	0.0535	1	0.0551	0.0185	1
VNII	10	7	1.5655***	0.5307***	1	0.8809**	0.3257**	1
VNIV	10	8	1.0722***	0.3643***	1	0.6535*	0.018*	1
VGI	10	9	1.0818***	0.3692***	1	0.9189**	0.3151**	1
VGII	13	10	0.4573**	0.1538**	0.6666	0.1194	0.0399	0.6666
VGIII	10	7	0.9015***	0.3038***	1**	0.4605	0.16059	1
VGIV	10	5	2.2432***	0.7641***	1**	2**	1**	1

Note: PhI, Phylogenetic incompatibility.

* P<0.05.

** P<0.01.

*** P<0.001

The third analysis, the phylogenetic incompatibility test only rejected the null hypothesis of random mating for the inclusive datasets of VGIII and VGIV (Table 5).

### Genetic variation of each locus

Overall, the *ACT1* gene was the most conserved locus (Table 6). Analysis of the sequence variation of each molecular type revealed that sequences of the VNIV and VGII clades were the most variable in *C. neoformans* and *C. gattii*, respectively (Table 6). In contrast to the overall comparable genetic variation of the *Cryptococcus* species complex, the *IDE* locus was exceptionally conserved among individual molecular types (Table 6). The overall genetic diversities of *C. neoformans* and *C. gattii* were similar (*P*<0.376), but the clades of *C. gattii* were more diverse than those of *C. neoformans* (Table 7). The genetic diversity between clades was higher for *C. gattii* (VGI-VGIV) than *C. neoformans* var. *grubii* (VNI and VNII) (*P*<0.001) and *C. neoformans* var. *neoformans* (VNIV) (*P*<0.038) (Table 7).

**Table 6:** Percent similarity representing the genetic variation among the haploid molecular types of the *Cryptococcus* species complex.

Locus		*ACT1*		*URA5*		*PLB1*		*IDE*	
Total Characters		*1062*		*525*		*1877*		*581*	
Constant Characters		%Total	%PUI	%Total	%PUI	%Total	%PUI	%Total	%PUI
*C. neoformans*	VNI	99.72	99.72	99.81	99.81	99.68	99.79	99.66	100
	VNII	100	100	99.43	100	100	100	98.97	100
	VNII-1	100	N/A	100	N/A	99.99	N/A	100	N/A
	VNIV	99.25	99.53	99.24	99.24	99.84	99.89	98.80	99.48
*C. gattii*	VGI	99.53	99.91	99.05	99.43	99.36	99.68	100	100
	VGII	99.34	99.44	98.29	99.43	98.88	99.04	99.83	100
	VGIII	99.81	99.81	99.24	99.62	99.63	99.79	99.83	100
	VGIV	100	100	100	100	99.89	99.95	100	100
	VGIV-1	99.99	N/A	99.99	N/A	99.99	N/A	99.98	N/A
Mean^*^		**99.66**	**99.77**	**99.29**	**99.65**	**99.61**	**99.73**	**99.58**	**99.93**
***C.s.*** complex^**^		**92.47**	**93.03**	**84.57**	**86.67**	**82.53**	**83.54**	**83.13**	**85.37**

Note: The VNII-1 and VGIV-1 PI were excluded due to limitation of the PAUP program. (^*^Mean = mean of variable percentage of all original molecular types. ^**^ *C.s.* complex = variable percentage calculated from the whole datasets. PUI = parsimony un-informative. N/A = not applicable).

**Table 7:** Number of polymorphic sites of the intron-excluded combined dataset among different clades of the *Cryptococcus* species complex.

Species and clades	Molecular type^*^	No. of polymorphic characters	No. of parsimony informative characters
***C. neoformans***		255	233
***C. n.*** ** var. ** ***grubii*** ** clade**		73	57
	VNI	12	7
	VNII	9	0
***C. n.*** ** var. ** ***neoformans*** ** clade**	VNIV	22	14
***C. gattii***		236	201
**VGI+VGIII+VGIV clade**		181	153
	VGI	24	10
	VGIII	14	8
	VGIV	2	1
**VGII clade**	VGII	38	27

Notes: ^*^VNII-1 and VGIV-1 were excluded. Genetic diversity (polymorphic characters) of *C. gattii* and *C. neoformans* is comparable (*P*<0.376). More genetic variation in the VGI+VGIII+VGIV and VGII clades, compared to the *C.n*. var. *grubii* (P<0.001) and *C.n.* var. *neoformans* clades (P<0.038), was observed.

### Evolutionary Divergence

**Figure 6:**
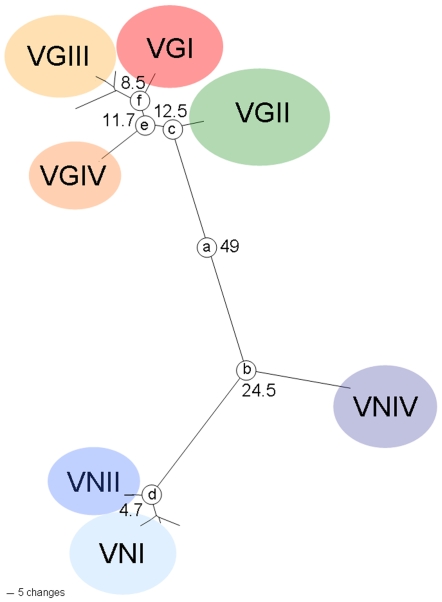
Genealogy of the intron-excluded combined dataset revealed the time since divergence among lineages of the *Cryptococcus* species complex. The number stated beside each node represents the ages of divergence in million years. Unlabelled branches are corresponding to VGIV-1 or VNII-1 groups, respectively.

Maximum-likelihood estimations with and without a molecular clock demonstrated that every gene did not differ significantly from molecular clock expectations (*P* = 0.99). Comparing the two species, the *C. gattii* lineages (12.5 million years ago) evolved later than the major *C. neoformans* lineages, *C. neoformans* var. *grubii* and *C. neoformans* var. *neoformans* (24.5 million years ago), suggesting more recent recombination events (Table 8 and Figure 6). However, the most recent speciation event took place around 4.7 million years ago splitting the two monophyletic lineages within *C. neoformans* var. *grubii,* VNI and VNII (Table 8 and Figure 6).

**Table 8:** Genetic distance and estimates of the times since divergence among gene lineages of the *Cryptococcus* species complex.

Between taxon/major molecular type group	Node	Genes					Time since divergence in million years (95% confidence interval)
		*ACT1*	*URA5*	*PLB1*	*IDE*	Averages** (95% confidence interval)	
*C.n. - C.g.*	a	0.0399	0.1042	0.1315	0.0910	0.0980 (0.0890–0.1071)	49.0 (44.5–53.5)
*C.n.* var. *grubii* – *C.n.* var. *neoformans*	b	0.0132	0.0426	0.0705	0.0506	0.0490 (0.0401–0.0579)	24.5 (20.0–28.9)
VNI – VNII^*^	d	0.0058	0.0088	0.0089	0.0185	0.0094 (0.0076–0.0113)	4.7 (3.8–5.7)
VGI – VGIII^*^	f	0.0083	0.0071	0.0262	0.0129	0.0171 (0.0133–0.0209)	8.5 (6.7–10.4)
VGI,VGIII – VGIV^*^	e	0.0145	0.0447	0.0262	0.0117	0.0235 (0.0196–0.0273)	11.7 (9.8–13.6)
VGI,VGIII,VGIV – VGII^*^	c	0.0099	0.0296	0.0326	0.0242	0.0221 (0.0221–0.0280)	12.5 (11.1–14.0)

Notes: ^*^ VNII-1 and VNIV-1 was not included in this calculation due to ambiguous placements in the molecular type lineages.

** weighted averages of the genetic distance of all genes combined.

### VNII-1 group is identical to the previously identified VNB molecular type according to parsimony and Bayesian analysis

**Figure 7:**
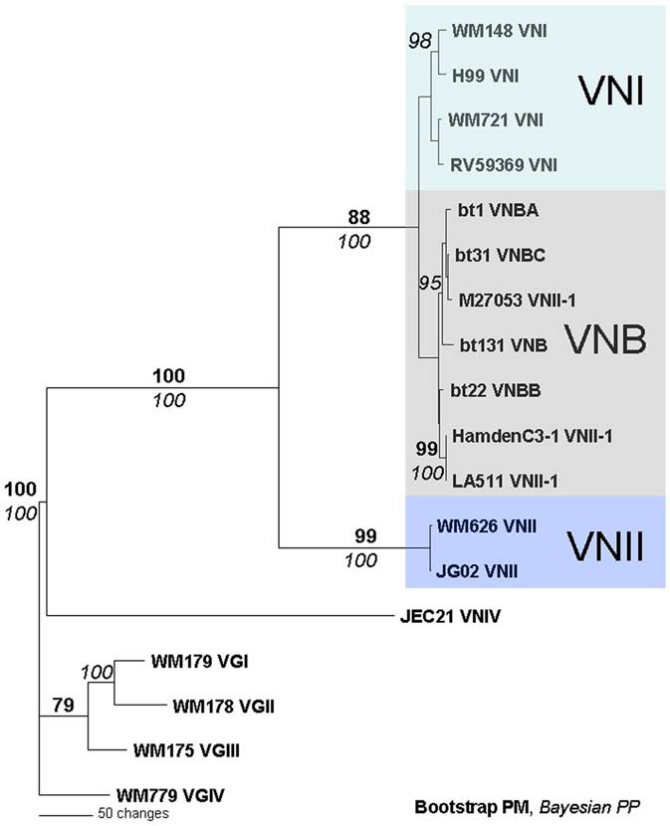
Genealogy of the combined dataset (*PLB1*, *URA5*, *GPD1*, *IGS1*) showing that the VNII-1 group isolates cluster with the VNB isolates previously described [15].

To determine whether VNII-1 isolates cluster with molecular type VNB, we performed a phylogenetic analysis with representative isolates of each molecular type including sequences of VNB isolates previously identified (bt1, bt31, bt131, bt22) [15]. The ILD test showed congruence for all pairs of the genes except *IGS1* when pairing the data with *URA5* and *GPD1* (*P*<0.0001). However, since the *IGS1* was used to describe the VNB molecular type [15], we included *IGS1* in the combined dataset. A heuristic search of the *ACT1* gene sequences found five maximum parsimony trees (Length 796, CI 0.884, RI 0.928). The tree topologies from the Bayesian analysis were similar to that found in the parsimony analysis. Despite the lack of significant support (<75 for parsimony bootstrap and <95 for Bayesian posterior probability), the VNII-1 strains were related to the VNB strains (Figure 7). Strain M27053 was closely related to strain bt31 with 100% support from the Bayesian posterior probability and 72% support from maximum parsimony bootstrap. The clade containing strain M27053 was significantly supported with a Bayesian posterior probability of 95% (Figure 7). The sequences were deposited in GenBank under the following accession numbers: *URA5* (EU929059–62), *GPD1* (FM180481–92), *IGS1* (FM180493–504).

## Discussion

The multigene phylogeny based on the *ACT1, URA5, PLB1* and *IDE* sequences provides an additional support for the currently accepted two species concept for the pathogenic *Cryptococcus* species complex (*C. neoformans* and *C. gattii*). The seven clades in the phylogenetic trees reflect the haploid molecular types recognized previously by M13 fingerprint, AFLP and RFLP analyses [8], [9]. For both species the obtained phylogenetic trees correspond to these molecular types and are highly supported by both maximum parsimony and Bayesian analyses. Clinical and environmental isolates clustered together in the respective major molecular types as previously determined in an analysis of six genetic loci (*ITS1/2, IGS1, CNLAC1, RPB1, RPB2* and *TEF1*) [14]. Here we found that the sequence diversity was comparable among strains of *C. neoformans* and *C. gattii* strains. Within the sample of *C. neoformans*, strains of *C. neoformans* var. *grubii* (VNI, VNII-1 = VNB and VNII) were more than twice as variable as strains of *C. neoformans* var. *neoformans*, confirming data reported in previous studies using different genetic loci and different sets of strains [8], [13]. MLST analysis of *C. neoformans* strains from sub-Saharan Africa had also revealed extensive genetic diversity among *C. neoformans* var. *grubii* strains [31].

Based on the four loci analyzed, *C. neoformans* and *C. gattii* share an equivalent level of molecular variation. Extensive surveys of the *Cryptococcus* species complex in South America have revealed extensive genetic diversity among strains of *C. gattii*, as well as the coexistence of both mating types in nature and evidence of recombination [9], [26], [32]. These points, taken together, suggest that the evolutionary origin of *C. gattii* may be in South America.

Occasionally, molecular typing based on M13 fingerprinting, AFLP and RFLP analysis is misleading. We obtained conflicting results when comparing strains that were typed using PCR-based (PCR fingerprinting, RFLP and AFLP) analyses and sequence-based methods. For example, strains of “VNII-1” were genotyped as VNII according to the *URA5* RFLP analysis but following multigene sequence analysis, they clustered with the VNI clade. Similarly, “VGIV-1” strains were identified as VGIV by *URA5* RFLP analysis but clustered with the VGIII clade on multigene sequence analysis. Moreover, *URA5*-RFLP analysis of the VNII-1 and VGIV-1 clades gave different molecular types from those obtained by *PLB1*-RFLP analysis (data not shown). These discrepancies may be attributable to insufficient markers, genetic drift or recombination events (Figure 5).

Similar to results presented by others [11], [12], our data indicate a lack of geographic concordance with the phylogeny, which suggest recent global dispersal of the *Cryptococcus* species complex. In addition, the apparent bias of *MATα* over *MAT* ***a***, as reported in previous surveys [33], [34], would limit the ability of the yeast to undergo sexual reproduction and recombination, which would retard speciation. Thus, clonal populations have been documented in several cryptococcal habitats, including Australia [35], [36], Thailand [37] and Canada [6] where the *MAT* ***a*** is scarce.

To investigate the question of recombination within the *Cryptococcus* species complex, we analyzed the congruence or incongruence of individual gene genealogies (ILD/PHT test) [38], [39] and the linkage disequilibrium (I_A_, rBarD and phylogenetic incompatibility) [38], [40] to infer the extent of clonality. Though clonal, asexual reproduction is the main reproductive mode in the *Cryptococcus* species complex, the existence of recombination and putative sexual reproduction has been demonstrated in several natural populations [31], [35], [41], [42]. In this investigation, the VGII clade was the only molecular type displaying unequivocal evidence of recombination in all the tests. Recent studies have provided evidence of sexual reproduction among strains of VGII on a global scale [43] and in local populations of Australia [35], [44]. Frequent recombination in the global population of VGII could explain the capacity of this genotype to expand its ecological range, as has been seen in the case of the Vancouver Island outbreak [6]. In contrast, the global populations of VGI, VGIV and VNII seem to be predominantly clonal. Previous analysis of AFLP and MLST data also found limited evidence for recombination for these molecular types [15], [35]. However, recent studies of selected populations found evidence for genetic re-assortment among environmental strains of VGI [45] and from veterinary strains of VNI [46]. For VNI and VGIII, we observed evidence of linkage equilibrium, but the ILD/PHT analysis data showed the presence of clonality. Among global isolates, our results reflect clonality for most molecular types within the *Cryptococcus* species complex except VGII, but they do not reject the possibility of sexual reproduction within any molecular type if sampled within a circumscribed population [e.g. 47]. The ILD/PHT *P* value for each gene combined individually (*P* values ranged from 0.010 for *ACT∶PLB1* to 0.835 for *URA5∶IDE*) and all genes combined (*P* = 0.002) showed no significant incongruence using a cut off point of 0.0001 [30], indicating a low degree or absence of recombination between the major molecular types, when only strains representative of the main lineages were used.

The multigene parsimony and Bayesian trees of the *ACT1* gene of *C. gattii* showed some incongruences in the topologies between the molecular types, indicating that more recent recombinational events may have occurred. This observation is consistent with the estimation that the clades of *C. gattii* diverged prior to those of *C. neoformans*, as indicated in Figure 6. This divergence time is approximately in agreement with previous studies, which used a different set of genetic loci [11], but closer to the recent estimations based on the whole genome, which estimated a split between the two species at 80 million years [48]. The time since divergence between each molecular type clade of *C. gattii* (11.7 million years between VGIV and VGI/VGIII and 8.5 million years between VGI and VGIII) is much more than between VNI and VNII (4.7 million years ago) (Figure 6). In addition, the divergence between VGII and VGI/VGIII/VGIV estimated to be 12.5 million years is in a similar range as the previously obtained divergence between the two varieties of *C. neoformans*, *C. neoformans* var. *grubii* and *C. neoformans* var. *neoformans* [11]. Hence, changing each clade to at least varietal status should be considered.

The VNB clade is recognized as a unique and highly diverse subpopulation of haploid isolates found in southern Africa [15]. Since the current study identified a special clade for the VNII-1 strains in the phylogenetic trees, which was in a similar position as the previously described VNB strains [15], [49], we conducted a preliminary phylogenetic analysis of the VNII-1 strains using four genetic loci, *PLB1*, *URA5*, *GPD1* and *IGS1*, and included a representative strain from each VNB sub-group: bt1 (VNB-A), bt22 (VNB-B), bt131 (VNB) and bt31 (VNB-C) [15]. The sequence analysis showed that the VNII-1 strains are closely related to the VNB clade (Figure 7). One of the strains, bt31, was almost identical to the strain M27053, which originated from South Africa. The other two isolates, Hamden C3-1 and LA511, which originated from South America (Brazil and Colombia, respectively), had related sequences, but without significant support from both analyses. However, these results suggest that VNB strains may not be unique to southern Africa. Although the clade containing strains Hamden C3-1 and LA511 did not receive significant support here, a previous study showed that strain Hamden C3-1 clustered with a VNB strain [14]. Considering their sequence similarities (Figure 5), VNB and VNII-1 strains may represent a link between the VNI and VNII clades. Whether VNB merits status as a distinct molecular type remains an open question, which needs to be addressed specifically in further studies with more VNB strains to be collected from Africa, South America and elsewhere. Bovers *et al*., 2008 have established a link, using MLST and AFLP analysis, between VNB isolates and their AFLP group 1A, where their AFLP group 1B clusters with isolates of the molecular type VNII [14]. *URA5*-RFLP analysis is not able to make this differentiation, where both AFLP groups, AFLP1A and AFLP1B, are associated with VNII, which includes the VNII-1/VNB isolates. Perhaps more importantly, DNA sequence markers are more reliable and discriminatory than AFLP genotypes. However, this report has also demonstrated that additional markers as well as additional strains are needed to resolve the number of legitimate clades of both *C. neoformans* and *C. gattii*.

A group of strains (VGIV-1) with an unusual clustering was also found in the *C. gattii* clade. The VGIV-1 strains clustered between VGIII and VGIV. To date, all the strains of VGIV-1 have been isolated in South America. If this geographic origin is substantiated, they perhaps represent a recombining population. Similar to VNB strains in southern Africa [31], there is a large population of fertile *MAT* ***a*** strains in South America [26], [50].

Two of those unusual clustering *C. gattii* strains (LA 390 and LA 392, both from Mexico) are the first VGIV strains identified as being *MAT* ***a*** **.** The mating type of the two strains has been identified by two independent mating type specific PCR, which amplified only the *MF* **a** pheromone gene and the *SXI2* **a** gene from both strains. The mating type was confirmed by mating experiments with the *C. gattii*, serotype C, *MATα* reference tester strain NIH312 [27] as well as with the *MATα* supermater strain JF101 [28] (Figure 2).

This study provided further information on the genetic diversity among members of the *Cryptococcus* species complex showing that it consists of at least seven monophyletic lineages, excluding the AD hybrid (VNIII) strains. The present study highly supports the two species concept recognized currently. The genetic variation found between the major molecular types of *C. neoformans* var. *grubii* is in the same range as the genetic variation found between *C. gattii* types. There are three monophyletic phylogenetic clades within the species *C. neoformans* two closely related lineages (VNI and VNII) observed in *C. neoformans* var. *grubii* and a basal lineage for *C. neoformans* var. *neoformans* (VNIV). The four molecular types of *C. gattii* (VGI, VGII, VGIII and VGIV) are highly supported by both maximum parsimony and Bayesian analyses. A similar topology was obtained previously investigating the intergenic spacer regions [12] and *PRP8* inteins [13], with high support (over 80%) from parsimony and Neighbor Joining analyses, respectively. The molecular type VGII forms the basal clade within the *C. gattii* branch of the phylogenetic tree and is phylogenetically more distant than the other three sibling clades, VGI, VGIII and VGIV. This result correlates with the finding of a specific mating characteristic for the VGII Vancouver Island outbreak isolates, which produced a basidium on stunted filaments close to the surface of the colonies when mated with other VGII strains [28]. The same morphology was also obtained by matings between other pairs of VGII isolates from different parts of the world [51]. This study revealed a level of genetic variability among the different molecular types/monophyletic lineages, which are comparable to, or in fact slightly greater than that found for *C. neoformans* var. *grubii* and *C. neoformans* var. *neoformans*.

The molecular clock analysis of the genetic diversity among the seven haploid major molecular lineages support the phylogenetic species concept, in which strains that form consistently the same monophyletic groups should be considered to be independent species [52], [53]. Especially the molecular types within *C. gattii* should be considered as varieties. However, the occasional hybrid strains that form between either of the two currently recognized species ([16], Trilles and Meyer pers. communication) or between the different monophyletic groups [54], [55] question the biological species concept [56], which defines a species as consisting of members that are able to mate and undergo sexual recombination. Therefore the question: whether there are more than two species within the *Cryptococcus* species complex or whether the molecular types/monophyletic lineages within each species deserve varietal status, remains open. A combination between the six recently published loci [14] and the four reported here would be desirable together with further morphological and mating studies to draw final conclusions.

## Materials and Methods

### Studied strains

Seventy three strains composed of at least ten representative strains of each haploid molecular type of the *Cryptococcus* species complex from different parts of the world, and the outgroup species *Cryptococcus albidus* were retrieved from the Molecular Mycology Research Laboratory culture collection at Westmead Hospital, University of Sydney, Westmead, NSW, Australia (Table 1). The four VNB strains were retrieved from the Culture Collection of the Department of Molecular Genetics and Microbiology, Duke University Medical Center, Durham, NC, USA. The hybrid molecular type, VNIII (serotype AD), was excluded from the study due to ambiguity of phylogenetic resolution in terms of variation in numbers of copies of each locus, and deserves to be studied separately [42], [49]. The strains were grown on Saboraud Dextose Agar (2% glucose, 2% peptone and 2% agar) for 72 hr before extracting DNA.

### DNA extraction

DNA extractions were performed by the liquid nitrogen grinding method as previously described [9]. Genomic DNA of the other outgroup *Filobasidiella depauperata* was kindly provided by Ferry Hagen (CBS – Fungal Biodiversity Centre, Utrecht, the Netherlands).

### Serotyping

The serotypes of the studied isolates were determined by the slide agglutination test using the Crypto Check Kit according to the manufactures instructions (Iatron Labs. Tokyo, Japan).

### Identification of the molecular type

*URA5*-RFLP analysis using the enzymes *Hha*I and *Sau*96I was performed initially to verify the molecular type of each studied strain as previously described [9].

### Determination of the mating type by PCR

Two pairs of mating-type specific primers were used. MFαU and MFαL primers identified the α-mating type (*MATα*) [57]. MFa2U and MFa2L recognized the **a-**mating type (*MAT* ***a***) [28] (Table 9). The *MAT* ***a*** strains of VGIII and VGIV were confirmed by sequencing the mating type specific amplicons and comparing them with the appropriate homologous sequences in GenBank uisng BlastN searches. *MAT* ***a*** of VGIV were additionally verified using a second set of mating type specific primers, SXI1αF and SXI1αR identifying *MATα* and SXI2**a**F and SXI2**a**R recognizing *MAT* ***a*** [44] (Table 9). The used PCR conditions have been published previously [28], [44], [57].

**Table 9:** List of primers used in this study.

Primers names	Primers sequences	Note	Source
CNACT1	5′ AATCTCGCCCAACATGT 3′	Amplify *ACT1*	This study
CNACT1R	5′ TTAGAAACACTTTCGGTGGACG3′	Amplify *ACT1*	This study
CNACT1F2	5′ CCAAGCAGAACCGAGAGAAG 3′	Internal primers of *ACT1*	This study
URA5	5′ ATGTCCTCCCAAGCCCTCGACTCCG 3′	Amplify *URA5*	[8]
SJ101	5′ TTAAGACCTCTGAACACCGTACTC 3′	Amplify *URA5*	[8]
PLBCNAF	5′ TAAAGTGCTTGGTGGGAACC 3′	Amplify *PLB1* from VN	This study
PLBCNAR	5′ TCTCGCGAGGATTACAGGAT 3′	Amplify *PLB1* from VN	This study
PLBCG2F	5′ TCCCCTTCAACACAGCTCTT 3′	Amplify *PLB1* from VG	This study
PLBCG2R2	5′ CACCTATCTTCGCTGCATCA 3′	Amplify *PLB1* from VG	This study
PLBCNIF1	5′ GGTTACCGTGCAATGCTGT 3′	Internal primers of *PLB1*	This study
PLBCNIF2	5′ GGTGCTTTCACCCCTATTGA 3′	Internal primers of *PLB1*	This study
PLBCNIR1	5′ CGGGAAATATCAGCTTGGTC 3′	Internal primers of *PLB1*	This study
IDEF	5′ CCAAGGCGGACAAGGCTGCGG 3′	Amplify *IDE*	[19]
IDER	5′ GTAGAGGTGATCCATGTCGGG 3′	Amplify *IDE*	[19]
ACT1CAF1	5′ GGTGTCATGGTCGGTATGG 3′	Amplify *ACT1* from CA	This study
ACT1CAR1	5′ GTACTTTCGCTCGGGAGGAG 3′	Amplify *ACT1* from CA	This study
ACT1CAR2	5′ AGCTTCTCCTTGATGTCTC 3′	Amplify *ACT1* from CA	This study
URA5DF1	5′ CCWTACTTCTTCAAYGCYGG 3′	Amplify *URA5* from FD	This study
MFLL	5′ CTTCACTGCCATCTTCACCA 3′	Mating type α determination of VN	[69]
MFLR	5′ GACACAAAGGGTCATGCCA 3′	Mating type α determination of VN	[69]
MFAL	5′ CGCCTTCACTGCTACCTTCT 3′	Mating type a determination of VN	[69]
MFAR	5′ AACGCAAGAGTAAGTCGGGC 3′	Mating type a determination of VN	[69]
MFαU	5′ TTCACTGCCATCTTCACCACC 3′	Mating type α determination of VG	[47]
MFαL	5′ TCTAGGCGATGACACAAAGGG 3′	Mating type α determination of VG	[47]
MFa2U	5′ ACACCGCCTGTTACAATGGAC 3′	Mating type a determination of VG	[38]
MFa2L	5′ CAGCGTTTGAAGATGGACTTT 3′	Mating type a determination of VG	[38]
SXI1αF	5′ TACATCACCGGTCATATCTGC 3′	Mating type α determination of VGIV	[68]
SXI1αR	5′ CTGGAGAAGCGCCTCACTGGA 3′	Mating type α determination of VGIV	[68]
SXI2aF	5′ TGATCGCACGAGCCAAATCCC 3′	Mating type a determination of VGIV	[68]
SXI2aR	5′ GGCTTCCTGACAACACTTCTA 3′	Mating type a determination of VGIV	[68]
GPD1F	5′ CCACCGAACCCTTCTAGGATA 3′	Amplify *GPD1*	[70]
GPD1R	5′ CTTCTTGGCACCTCCCTTGAG 3′	Amplify *GPD1*	[70]
IGS1F	5′ ATCCTTTGCAGACGACTTGA 3′	Amplify *IGS1*	[14]
IGS1R	5′ GTGATCAGTGCATTGCATGA 3′	Amplify *IGS1*	[14]

Notes: VN = molecular type VNI, VNII and VNIV; VG = molecular type VGI, VGII, VGIII and IV, FD = *Filobasidiella depauperata*, CA = *Cryptococcus albidus*.

### Mating

The fertility of *MAT* ***a*** VGIV strains was investigated by mating them with the following *MATα* and *MAT* ***a*** reference tester strains: *C. gattii* clinical, serotype C, strains NIH312 (*MATα*) [27] and B4546 (*MAT* ***a***) [29], and *crg1*α mutant derivatives (“supermater” tester strains), JF101 (*MAT*α) and JF109 (*MAT* **a**) [28]. The *MAT* ***a*** VGIV strains (LA390 and LA392) reported herein were co-cultured with each reference tester strain. Cells from each mating partner were mixed on V8 juice agar (5% [vol/vol], 3 mM KH_2_PO_4_, 4% [wt/vol] agar, adjusted to pH 5 [58]) and incubated in darkness for up to four weeks. Sexual reproduction was confirmed by the presence of chains of basidiospores and fused clamp connections. All strains were individually incubated on V8 juice agar as described above to differentiate haploid fruiting from mating.

### Chromosomal location of the gene loci studied

Four independent genetic loci were used in the multigene sequence analysis. The chromosomal location for each of those genes was determined via BlastN searches against the genome of the serotype D strains JEC21 [59] in GenBank. The *ACT1* locus is located on chromosome 1, the *PLB1* locus is located on chromosome 13, the *URA5* locus is located on chromosome 7 and the *IDE* locus is located on chromosome 12.

### Gene amplification and sequencing

The *ACT1* [17], *URA5* [3], *PLB1* [18] and *IDE* [19], [22] genes were amplified from each strain using specific primers (see Table 9). Each PCR contained 50 ng of genomic DNA, 50 ng of each primers, 0.2 mM dNTP, 3 mM MgCl_2_ and 0.5 unit of either AmpliTaq® (Applied biosystems, CA, USA), BioTaq® (Bioline, NSW, Australia) or ExTaq® (Takara, Shiga, Japan) DNA polymerase with 1X of compatible buffer in a total volume of 50 µl. PCR conditions for the *ACT1* gene amplification were as follows: 3 min of initial denaturation at 94°C, followed by 35 cycles of 45 seconds at 94°C, 45 seconds at 56°C, 1 min at 72°C and terminating with 7 min of final extension at 72°C. Amplification conditions of the other genes were similar to the protocol for *ACT1*, but the annealing temperatures and extension times differed, as follows: *URA5* 62°C and 2 min; *PLB1* for strains of VNI, VNII and VNIV 60°C and 2 min; *PLB1* for strains of VGI, VGII, VGIII and VGIV 58°C and 2 min; and *IDE* 62°C and 1 min. *ACT1* of *F. depauperata* was amplified directly using the primers CNACT1 and CNACT1R whereas and the *ACT1* of *C. albidus* was amplified using the primers ACT1CAF1 and ACT1CAR1 (Table 9). *URA5* of *F. depauperata* was amplified using the primers URA5DF and SJ101 (Table 9). To resolve non-specific bands generated by several *URA5* amplifications of *F. depauperata* we cloned the amplicons using the pGEM®-T Easy Vector System I according to the manufacturer's protocol (Promega®, NSW, Australia). PCR amplicons and the *URA5* plasmid were purified and sequenced by the ABI Big dye Terminator method (Macrogen Inc., Korea) using the amplification and additional internal primers (Table 9). Sequences were assembled using either the program Sequencher version 4.6 (Gene Codes, MI, USA) or Bioedit version 7.0.5.3 [60]. Intron and exon positions were determined by aligning the sequences with the reference sequences of each gene as follows: *ACT1* (GenBank Accession No. U10867), *URA5* (GenBank Accession No. AF032436), *PLB1* (GenBank Accession No. AF223383), *IDE* (GenBank Accession No. XM568105). Sequences including and excluding introns were aligned with Bioedit version 7.0.5.3 [60] using Clustal W [61]. Sequences were deposited in Genbank (see below). Combined datasets were created by a combination of the *ACT1*, *URA5*, *PLB1* and *IDE* sequences. Sequences from the closet siblings of the pathogenic *Cryptococcus* species complex: *F. depauperata and C. albidus* were used as outgroups [1], [62].

### Phylogenetic analyses

The phylogenetic relationships within the *Cryptococcus* species complex were inferred by parsimony and likelihood methods. Parsimony analysis was conducted with the program PAUP* 4.0b10, using the heuristic search option [63]. For the maximum parsimony analysis starting trees were obtained by stepwise addition with 100 random sequence additions. Tree bisection-reconnection (TBR) was used for branch-swapping. Maximum parsimony phylograms were generated for each of the four loci, as well as the combined datasets, which included every locus of all the isolates. Bootstrap analysis using 500 heuristic replicates was used to estimate support for the clades of each locus with MaxTree set to 100. Gaps in the sequences were treated as missing data and all characters were equally weighted.

Bayesian phylogenetic analysis was used to estimate the probability of the taxonomic structure given the data at each individual locus, as well as the combined data from all four loci using MrBayes 3.1.2 [64]. First, the model of nucleotide substitution that best fit the data of each locus was determined by a likelihood ratio test using the program PAUP* 4.0b10. The likelihood score files, created in PAUP, were then analyzed with the program ModelTest 3.7 on the ModelTest server 1.0 (http://darwin.uvigo.es/software/modeltest_server.html) using the Akaike information criterion (AIC) [65]. In the analysis of the combined loci, parameter estimates of each locus were unlinked, allowing independent substitution models for each locus. Two analyses were performed simultaneously and used to calculate the posterior probabilities, as estimated from uniform priors, of the clades of each locus and the combined loci. Each analysis included four simultaneous and incrementally heated Markov chains; each replicate used default heating values. Markov chains were initiated from a random tree and were run for 1,000,000 generations. Samples were taken every 100^th^ generation. Standard deviations of split frequencies of the two runs were monitored until they converged. The last 5,000 samples of each analysis containing the standard deviations ≥0.02 were used to generate consensus trees using PAUP under the 50% majority rule. The clade posterior probabilities of each clade and the overall topology of each replicate were compared to verify that each consensus tree converged on a similar phylogeny.

### Sequence similarity determination

The sequence similarity matrix among the major haploid molecular type clades was created by the program MEGA version 4.1 [66] based on the intron-included dataset. P-distances were generated and converted to similarity percentage using Microsoft Excel program. Standard errors were computed using 500 bootstrap replicates.

### Combinability assessment

Prior to combined analyses the combinability of the data were explored using incongruence length difference/partition homogeneity test (ILD/PHT) [67], [68] implanted in PAUP. To avoid detecting incongruence that is expected within lineages (see below), IDL/PHT was restricted to datasets containing only single reference strains representing the major haploid molecular types and the major lineages obtained by the phylogenetic analyses of the *Cryptococcus* species complex (strains: ATCC90112, WM148, M27053, WM626, HamdenC3-1, RV58146, WM629, JEC21, WM179, MCS022, WM178, RB52, WM175, LA644, WM779, M27055, LA390 and LA568). The ILD/PHT analysis used only informative characters and entailed a random stepwise-addition maximum parsimony heuristic searches with 10000 replicates (TBR; maxtrees = 500). Since a significance threshold of 0.05 forces the ILD/PHT computation to be too conservative [30], the null hypothesis of congruence was rejected only if *P*<0.0001.

### Recombination assessment

To determine the extent of recombination, if any, within each molecular type, we implemented three complementary tests: the ILD/PHT test, the Index of Association (I_A_) and the phylogenetic incompatibility.

In the first test, we used the ILD/PHT with the objective to find signals of recombination in our dataset. In the absence of recombination, all the loci should provide the same phylogenetic result and therefore all the gene trees should be compatible and alleles at different regions should be associated. In contrast, in a recombining population each locus is expected to have a unique evolutionary history resulting in a different phylogeny for each locus thus this phylogenies are incongruent and incompatible. This test was conducted using PAUP including all the isolates under the same conditions explained previously.

In the second test we used the I_A_, that is the most common measure of multi-locus linkage. The I_A_ uses an estimate of multi-locus association within a haploid population through assessment of the number of loci that are different when individuals are compared. The expected difference between a recombinant population and a clonal one is that a clonal population will have a skewed distribution with a statistically significant excess of extreme distances, whereas a recombinant population will have a genetic distance with a normal distribution [47], [69]. In view of the fact that the number of loci analyzed can influence the expected value of the I_A_ we complemented these analysis using the statistic rBarD that is a modification of I_A_, which removes this dependency on number of loci. For I_A_ and rBarD analysis, we used the program MultiLocus 1.3 [70] (http://www.agapow.net/software/multilocus/) using 1000 artificially recombining datasets. The null hypothesis of recombination is rejected if the I_A_ or rBarD of the observed dataset is significantly (*P*<0.05) different than the range of values produced from the artificially recombined datasets.

In the third test, we calculated the proportion of pairwise loci that were phylogenetically incompatible. Two loci are compatible if all the observed genotypes are explainable by mutation rather than recombination or homoplasy [71]. For example, if there are two loci with two alleles each, the two loci are incompatible if all four possible genotypes can be found in the population, showing a possible genetic exchange between strains. This test was performed using the program Multilocus 1.3 using 1000 artificially recombining datasets. *P* values of less than 0.05 indicate that the hypothesis of random recombination should be rejected.

### Genetic variability analysis

Genetic variations of each locus were determined by using the program PAUP* 4.0b10. The variation among all sequences, as well as the parsimoniously informative characters, was calculated from each reference molecular type strain and from all cryptococcal strains.

Numbers of polymorphic characters of the combined loci from the intron-excluded dataset of each clade were compared using the Chi-Square test. The statistical analysis was performed with the program SPSS 15.0.0 (LEAD Technologies, Inc., IL, USA) and significance was defined when *P* value<0.05.

### Estimation of genetic divergence among lineages

Genetic divergence among each lineages was estimated as previously describe [11] with modification. A maximum-likelihood tree for each gene was created based on the selected evolutionary models with and without molecular clock and the Likelihood Ratio Test statistic was calculated. Chi-square test was performed to estimate whether the four genes evolved according to the molecular clock model (*P* value>0.05). Distances according to the selected evolutionary model for each gene were calculated from the maximum-likelihood tree [63]. Estimates of the time of divergence and hybridization assumed the consensus mutation rate of 2×10^−9^ per nucleotide per year for protein coding genes [11], [72], [73]. The distance was calculated between the most recent nodes of the hybrid to the tip of the phylogeny. Weighted average and standard deviation of the distance were calculated using the program SPSS 15.0.0 (LEAD Technologies, Inc., IL, USA). Among the four genes, the estimates of age show that the earliest hybridization event in each isolate is the maximum age of hybridization. The 95% confidence intervals were calculated using the program Excel (Microsoft Corporation, USA).

### Investigation of the relationship of VNII-1 group and the VNB molecular type

A recent study in Botswana has proposed a new molecular type, VNB for the *Cryptococcus* species complex [15]. We determined whether our VNII-1 isolates are in fact resided in this new molecular type. Parsimony and Bayesian analysis were performed as described above, using a combined dataset of *PLB1*, *URA5*, *GPD1*, and *IGS1* with representatives of each molecular type including: VNI: WM148, H99, WM721, RV59369; VNII: WM626, JG02; VNII-1: M27053, HamdenC3-1, LA511; VNB: bt1, bt31, bt131, bt22; VNIV: JEC21 and the standard molecular type strains of *C. gattii*. Bayesian analysis was performed based on HKY evolutionary model with the gamma distribution according to the program ModelTest 3.7 using the combined dataset obtained as described above. The combinability as tested as described above. Sequences of *PLB1*, *GPD1* and *IGS1* of the VNB strains were obtained from the online database (http://www.mlst.net/). Gene sequences of strain H99 were obtained from the genome project database (http://www.broad.mit.edu/) as well as *GPD1* and *IGS1* sequences of JEC21 (http://www.ncbi.nlm.nih.gov/). *URA5* of the VNB strains were amplified and sequenced as described above. *GPD1* and *IGS1* of the other strains were amplified using the primers GPD1F/GPD1R [68] and IGSF/IGSR [15] under the following conditions: 3 min of initial denaturation at 94°C, followed by 35 cycles of 45 seconds at 94°C, 45 seconds at 63°C (*GPD1*) or 60°C (*IGS1*), 1 min at 72°C and lastly, 7 min of final extension at 72°C.
