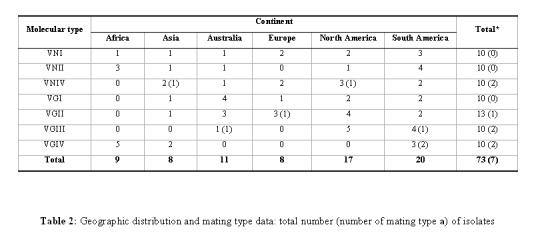# Correction: Genetic Diversity of the *Cryptococcus* Species Complex Suggests that *Cryptococcus gattii* Deserves to Have Varieties

**DOI:** 10.1371/annotation/3037bb69-1b8e-4d99-b169-afdf4b74ace2

**Published:** 2009-07-15

**Authors:** Popchai Ngamskulrungroj, Felix Gilgado, Josiane Faganello, Anastasia P. Litvintseva, Ana Lusia Leal, Kin Ming Tsui, Thomas G. Mitchell, Marilene Henning Vainstein, Wieland Meyer

The published Table 2 is an incorrect version. Please see the correct table here: 

**Figure:**