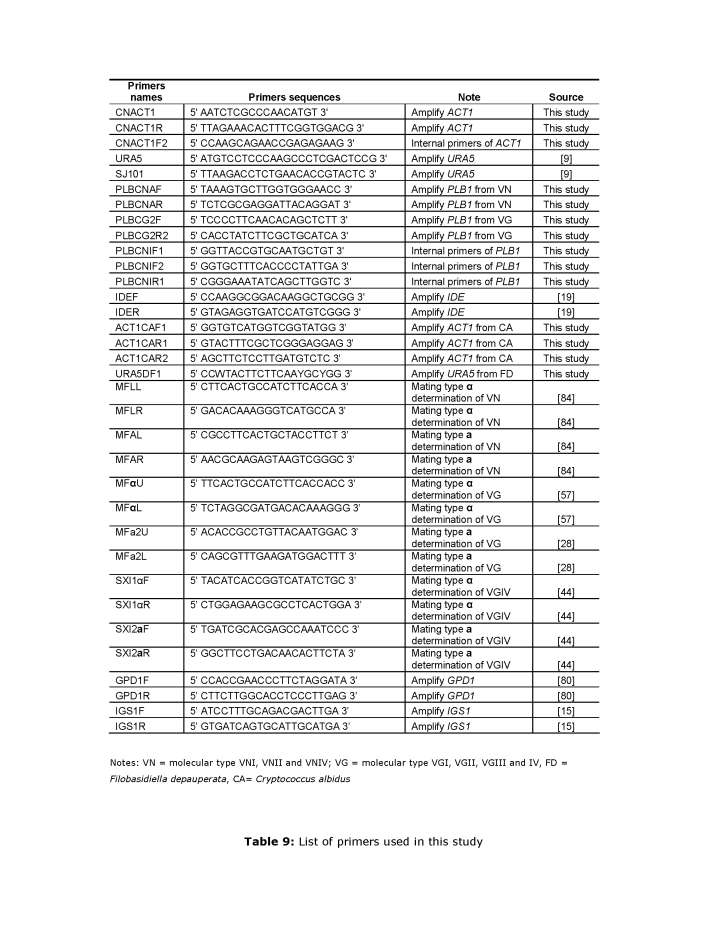# Correction: Genetic Diversity of the *Cryptococcus* Species Complex Suggests that *Cryptococcus gattii* Deserves to Have Varieties

**DOI:** 10.1371/annotation/348c3375-3918-4e41-bb8c-27aa15d2bdc4

**Published:** 2009-07-15

**Authors:** Popchai Ngamskulrungroj, Felix Gilgado, Josiane Faganello, Anastasia P. Litvintseva, Ana Lusia Leal, Kin Ming Tsui, Thomas G. Mitchell, Marilene Henning Vainstein, Wieland Meyer

Several references in the rightmost column of Table 9 are incorrect. Please see the corrected table here: 

**Figure:**